# Effect of a pocket size guideline book in the emergency department; a questionnaire study

**DOI:** 10.1186/1757-7241-21-S2-A23

**Published:** 2013-09-09

**Authors:** Melanie Correia Schrøder, Dan Brun Petersen

**Affiliations:** 1 Emergency Department, Holbaek University Hospital, Denmark

## Background

Traditionally Danish clinical guidelines cover specific diagnoses. However these guidelines are not always suitable for the acute patients presenting at the Emergency Department (ED). In 2009 the ED at Kolding Hospital developed a pocket size guideline book containing the conditions most commonly encountered in the ED. At Holbaek University Hospital guidelines are developed by each specialty and are only available online at the hospital intranet. We wished to investigate the effect of a pocket size guideline book.

## Methods

A questionnaire was sent to all junior doctors at the two EDs asking how easy it was to find the guidelines. Each question should be answered on 41 different subjects, mostly clinical but also administrative.

## Results

Almost all doctors from Kolding answered ”easy” or ”very easy” to finding most of the guidelines, but the return rate was only 7 out of 22 (32%), and consequently no comparison could be made.

The return rate from Holbaek was 11 out of 15 (73%). All answered “easy” to finding some guidelines and “difficult” to finding others, rendering no clear conclusion.

The majority answered that it is “easy” or “very easy” to find guidelines for Cardiac arrest (11/11), DVT (10/11) and Triage (10/11). On the contrary, it is ”difficult” or ”very difficult” to find guidelines for ECG (8/11), Involuntary Treatment of Psychiatric Patients (8/11), Fluid & Electrolyte treatment (7/11), and Pain Management (7/11).

One respondent commented: ”Information is easily found on the internet – not on the local intranet because of its inadequate search function”.

## Conclusion

We could not compare the two departments. However there is a great difference in how easily doctors can retrieve guidelines, which jeopardizes the use of the valid, local guidelines. Certain subjects need more attention than others.

A pocket size guideline book will be introduced in Holbaek in the spring of 2013. All junior doctors rotate out of the department every semester, thus after the next rotation we will repeat the questionnaire in order to investigate if the new group of doctors experience easier access to the local guidelines.

